# Maternal Psychological Control and Children's Sociobehavioral Adjustment Problems in European American, African American, and U.S. Mexican Families

**DOI:** 10.1007/s10826-025-03236-3

**Published:** 2026-02-12

**Authors:** Afra Agalar, Deborah Laible, Jihee Im, Clare Van Norden, Alysia Cruz, Gustavo Carlo, Jean Ispa

**Affiliations:** 1 School of Humanities and Social Sciences, Indiana University Kokomo, 2300 S. Washington St., Kokomo, IN 46902 USA; 2 Department of Psychology, Lehigh University, Chandler-Ullmann Hall, 17 Memorial Dr E, Bethlehem, PA, 18015 USA; 3 Population Research Institute, Pennsylvania State University, 315 Susan Welch Liberal Arts Building, University Park, PA 16802 USA; 4 Department of Psychology, Douglas College, PO Box 2503, New Westminster, BC V3L5B2 Canada; 5 School of Education, University of California, Irvine, 3200 Education Bldg, 92697 Irvine, CA USA; 6 Department of Human Development and Family Studies, University of Missouri, 102 Gwynn Hall, Columbia, MO 65211 USA

**Keywords:** Maternal psychological control, Guilt inductions, Shame inductions, Externalizing behaviors, Social adjustment, Ethnic and racial groups

## Abstract

Much of the research indicating that U.S. parents’ use of psychological control is connected to adverse child outcomes overlooks the specific dimensions of psychological control, relies on self-report measures and excludes ethnic and racial minorities. This observational study takes a step towards identifying the links between mothers’ use of guilt and shame inductions and children’s externalizing behaviors and social adjustment problems within European American, African American and U.S. Mexican families. A total of 436 children (49% female, *M*_age_ = 10.51 years) and their mothers from low-income households, representing these distinct ethno-racial groups, engaged in discussions on three conflict topics. Maternal guilt and shame inductions during conflict conversations were coded, and mothers completed measures of externalizing behaviors and social adjustment problems. Results showed that maternal shame induction was related to both externalizing behaviors and social adjustment issues, while guilt induction was related solely to externalizing behaviors. However, no significant differences were found across the ethno-racial groups in how guilt and shame inductions were related to children’s social and behavioral adjustment. These results highlight that two components of psychological control, guilt and shame inductions, are related to child outcomes in different ways and underscore the pervasive challenges that maternal psychological control presents for child development across ethno-racial backgrounds.

## Highlights

This observational study examined the associations between maternal guilt and shame inductions and child outcomes within African American, U.S. Mexican, and European American families.

Maternal shame inductions were positively associated with both externalizing behaviors and social adjustment problems.

Maternal guilt inductions were positively associated with externalizing behaviors only.

The associations between maternal guilt and shame inductions and children’s social and behavioral problems did not differ significantly across ethno-racial groups.

Mother-child discourse is an interactive process that holds significant implications for children’s socioemotional development (Eisenberg et al., 2008; Laible & Thompson, 2000). The way parents communicate and interact with their children can profoundly impact children’s adjustment and overall well-being. Employing positive discourse styles that involve inductive reasoning, elaboration, and support while validating the child’s perspective contributes to the development of children’s socioemotional competence (Laible & Thompson, 2002; Laible et al., 2015; Waters et al., 2010). In contrast, the use of psychological control tactics during mother-child discussions by manipulating children’s thoughts and feelings hinders children’s autonomy and independent expression. As a result, cultivating a healthy perception of self becomes challenging due to various factors. These factors include the indirect devaluation of the child, the absence of constructive engagement with others, a process that is instrumental in shaping a well-defined self-concept the limited opportunities to nurture a belief in one’s own capability to achieve goals, and disruptions in the process of self-exploration required for forming a stable and coherent identity (Barber, 1996; Marcia, 1980; Peterson & Seligman, 2004; Youniss & Smollar, 1987). Psychological control, then, poses a potential harm to children’s psychosocial functioning (Barber et al., 2005; Kuhn & Laird, 2011; Lansford et al., 2014).

Given that late middle childhood is marked by an increasing desire for autonomy, examining maternal psychological control during this period is especially important. Despite children’s growing independence during this period, they remain highly responsive to maternal influence (Pettit & Laird, 2002; Wray-Lake et al., 2010). Thus, how mothers navigate this developmental transition can significantly affect parent-child interactions and children’s well-being (Ispa et al., 2015; Yan et al., 2017). It is particularly important to examine psychological control in low-income families, where higher levels of control are frequently observed (Xu et al., 2021), likely due to the added stressors such as financial instability and limited resources. These socioeconomic challenges can shape how mothers interact with their children and ultimately impact their developmental outcomes (Grolnick & Apostoleris, 2002).

## Guilt and Shame Inductions

The act of inducing guilt and shame in children by parents is considered a form of psychological control (Barber et al., 2005). Even though guilt and shame inductions are sometimes viewed as a component of moral socialization in Asian cultures, much of the research on this topic, reviewed here, has focused on European American samples, where these parenting practices are generally linked to negative outcomes (Fung et al., 2003; Tangney et al., 2014). These outcomes are often attributed to the impact of guilt and shame on autonomy and independence, qualities that are highly valued in Western cultures (Barber et al., 2005; Donatelli et al., 2007). However, the influence of these practices may differ in other cultural contexts, particularly among minority populations, as discussed in a later section.

Shaming is thought to increase individuals’ self-consciousness regarding the visibility of their actions, as well as their awareness of external social expectations and the criticisms of others. As a result, experiencing shame can engender personal distress, particularly in the context of interpersonal conflicts (Ferguson & Stegge, 1998; Leith & Baumeister, 1998). The constant awareness of external judgments and expectations may impede an individual’s ability to express themselves authentically, and hinder the development of socially adept behaviors necessary for meaningful interpersonal connections. Moreover, shaming can evoke feelings of inferiority and reinforce a sense of inadequacy and worthlessness (Eisenberg, 2000; Lewis, 1971). These negative self-perceptions can lead individuals to approach social interactions with hesitancy and self-doubt and to perceive themselves as undeserving of acceptance and validation from others. Consequently, shaming can hinder children’s ability to navigate social situations effectively and pose challenges in forming and maintaining healthy relationships (Tangney et al., 1995). Furthermore, the induction of shame has the potential to elicit feelings of hostility and anger stemming from the perception of being placed in an inferior position. Due to the awareness of disapproval from others that accompanies shame, this anger can easily be directed towards others, resulting in the display of externalizing behavior problems (Lewis, 1971). Responding to shame with externalizing behaviors can serve as a defense mechanism aimed at safeguarding an individual’s positive self-regard and regaining the feeling of autonomy (Tangney et al., 2014; Thomaes et al., 2007). Ultimately, the use of shame inductions may negatively affect children’s social adjustment and lead to an increase in externalizing behaviors.

There is an ongoing debate regarding the impact of guilt induction on children’s social adjustment. Some scholars argue that guilt induction, which involves critiquing a specific behavior to evoke feelings of tension, remorse, and regret (Tangney et al., 1996), serves as a valuable tool for parents to educate their children about the consequences of their actions (Baumeister, 1998). The feeling of guilt may motivate children to take reparative action and engage in behaviors such as apologizing or rectifying the harm caused (Tangney et al., 1996). Consequently, proponents of this view suggest that instilling feelings of guilt in children can foster positive social interactions, including the cultivation of prosocial behavior and empathy (Eisenberg, 2000; Estrada-Hollenbeck, 1998; Hoffman, 2000). However, parenting literature also highlights negative associations between parental guilt induction and children’s social adjustment. Previous studies showed that guilt induction was linked to socioemotional problems such as difficulties in individuation and adjusting to new situations, less effective coping mechanisms, feeling anxious about being apart from parents and lack of autonomy in peer interactions (Hare et al., 2015; Mayseless & Scharf, 2009). Children may find the experience of guilt to be overwhelmingly distressing. This distress can lead to a cycle of negative feelings and self-blame for perceived shortcomings (Bybee & Quiles, 1998). Feeling burdened by guilt, children may struggle with self-esteem and confidence in social settings (Rudy et al., 2014). Furthermore, this distress can influence their ability to regulate emotions effectively, which leads to disruptions in social interactions (Luebbe et al., 2014). Despite the close interconnection between feelings of guilt and shame, shame exhibits a stronger association with indicators of maladjustment in children (Tangney et al., 1995).

These mixed findings about the consequences of guilt induction may be partly explained by the context in which guilt induction occurs and children’s developmental stage. When guilt induction takes place during parent-child conflicts, the existing relational tension is already high, which can significantly influence how children react (Eisenberg et al., 2008; Kho et al., 2022). Rather than processing guilt induction as a form of guidance, children may respond with feelings of pressure or distress. This heightened state may cause them to focus on immediate negative feelings, which diverts their attention from the intended lesson. As a result, they may feel controlled instead of supported, which may undermine the potential positive impact of guilt induction. Moreover, children’s perceptions and responses to guilt induction evolve with age. As children grow older and their need for autonomy strengthens, they tend to view guilt induction as increasingly intrusive and disrespectful, often attributing it to more negative and self-focused parental intentions (Rote & Smetana, 2017). This developmental shift may contribute to the positive association between guilt induction and social adjustment problems.

Despite the lack of existing research connecting maternal guilt inductions to an increase in children’s externalizing behavior, previous studies have demonstrated a positive association between broadly defined parental psychological control and externalizing behavioral issues in children (Bai et al., 2020; Gugliandolo et al., 2015; Lansford et al., 2014; Symeou & Georgiou, 2017). It is plausible that guilt induction occurring during conflict situations may lead children to reject parental messages and respond with heightened anger. These negative emotional reactions, in turn, may contribute to the development or intensification of externalizing behaviors. Further research is needed to better understand the link between parental guilt induction during conflict and children’s externalizing behaviors.

Many studies focusing on parental psychological control and child outcomes rely on self-report measures provided by parents or adolescents (Barber & Harmon, 2002; Soenens & Vansteenkiste, 2010). It is essential to acknowledge the limitations of self-report measures when interpreting results, as some conflicting findings may stem from these measurement issues. More observational research is necessary to understand the way mothers employ psychological control in real-life settings and its impact on children. Directly observing mothers’ behavior would allow researchers to minimize bias and obtain more accurate information about maternal psychological control. Moreover, psychological control is a multidimensional construct without consensus on each dimension (Barber et al., 2012; Fung & Lau, 2012). Some forms of psychological control may consistently negatively affect child development, while others may not. Therefore, considering specific dimensions of psychological control separately, such as guilt and shame inductions, is crucial for understanding their nuanced impact on children’s well-being.

## Maternal Psychological Control in African Americans and U.S. Mexicans

Existing research connects maternal psychological control to socioemotional issues, due to its negative impact on children’s autonomy, independence, and exploration (Barber et al., 2005; Donatelli et al., 2007; Kuhn & Laird, 2011; McKee et al., 2014; Tangney et al., 1996). However, much of this research focuses on majority populations in Western societies and often neglects the perspectives of ethnic and racial minorities within these societies. Moreover, the few studies that do address ethnic and racial minorities in the U.S. generally explore the broad concept of psychological control, rather than examining its specific aspects.

Minority parents in the U.S. may feel compelled to exercise parental control as a way to guide and protect their children, given the challenging circumstances of poverty, single parenting, limited educational opportunities, and systemic inequalities that disproportionately affect minority children and adolescents (García Coll & Pachter, 2002). Thus, parental control may be more commonly observed or considered normative in ethno-racial minority families. Indeed, existing research suggests that African American mothers, as compared to European American mothers, may exhibit higher levels of authoritarian parenting, which can include elements of psychological control (e.g., Burchinal et al., 2011; LeCuyer & Swanson, 2017; Ng et al., 2014). Moreover, African American families’ emphasis on respect for authority figures, balancing individual rights with group needs, and a strong sense of family duty and commitment to kin (Hill, 1972) may suggest that parental control may have different meanings or effects for African American children. Research shows that African American youth view guilt-based parental control positively and associate it with love and care (Mason et al., 2004). While some studies of African American youth found no association between maternal psychological control and negative outcomes including depression and antisocial behavior regardless of socioeconomic status (SES) (Bean et al., 2006), others linked it to lower self-esteem (Plunket et al., 2016) and higher levels of externalizing behavior problems (Kincaid et al., 2011). Given that the last two studies did not account for SES as a statistical control, any cross-cultural group differences between African American findings and those reported in European American samples may be due to variations in SES.

Similar to African American cultural contexts, Latine cultural contexts are characterized by a collectivistic and interdependent orientation with significant importance attributed to familism, education, and adherence to authority figures (Knight & Carlo, 2012; Tseng, 2004). Due to the normative expectation of interdependence, maternal psychological control may be more common and accepted compared to European American families and often seen as an expression of love and concern (Halgunseth et al., 2006). Studies show that parental psychological control in Latine families is perceived as part of positive parent-adolescent relationships and is linked to parental concern (Crockett et al., 2007). U.S. Mexican mothers aim to foster responsible behavior through a combination of psychological and behavioral control, alongside autonomy support (Yau & Watkins, 2018). U.S. Mexican adolescents see parental autonomy support and psychological control as intertwined (Sher-Censor et al., 2011). Thus, parental psychological control does not necessarily imply reduced autonomy or lack of parental care in this cultural context (Sher-Censor et al., 2011; Yau & Watkins, 2018). Limited research exists on the associations between parental psychological control and children’s outcomes within U.S. Mexican families. Perceived maternal psychological control has been found to be positively associated with depressive symptoms in U.S. Mexican adolescents, but not with self-esteem (Kline et al., 2016; Sher-Censor et al., 2011). Also, it has been positively related to anxiety in European American children but inversely related in Mexican American children (Luis et al., 2008).

The inconsistencies in findings regarding parental psychological control and child outcomes across ethno-racial groups may stem from the use of self-report measures which were primarily developed from a Western perspective (Barber & Harmon, 2002; Soenens & Vansteenkiste, 2010). These measures might not effectively capture the cultural nuances and variations of psychological control, especially regarding guilt and shame inductions that may vary across cultural contexts (Fung & Lau, 2012). There is a pressing need for empirical investigations, incorporating observational research that can provide a relatively naturalistic approach to examine the link between parental psychological control and children’s outcomes within European American, African American, and U.S. Mexican families.

## Current Study

The primary objectives of the current study were threefold: (1) to examine the relations between maternal guilt and shame inductions and children’s externalizing behaviors and social adjustment problems in a diverse sample by using observational methods, (2) to investigate ethnic and racial variations in these associations, and (3) to explore the extent to which mothers from European American, African American, and U.S. Mexican ethno-racial groups utilize these two aspects of maternal psychological control during conflict conversations. The study recognized the need for further research to understand how mothers utilize guilt and shame inductions to socialize children in real-life interactions across diverse ethno-racial groups. Notably, a significant majority of prior research has relied on self-reports from either mothers or children, with limited use of observational methods. By incorporating observational techniques, this study aimed to address this gap in the existing literature and provide valuable insights into the topic.

The hypotheses posited that both maternal guilt and shame inductions would predict children’s externalizing behaviors and social adjustment problems. Additionally, it was expected that these positive associations between mothers’ use of guilt and shame inductions and children’s externalizing and social adjustment problems would be stronger in European American families compared to African American and U.S. Mexican families. It was predicted that ethno-racial minority mothers would utilize guilt and shame inductions more than European American mothers. It was challenging to make predictions regarding the differences between African American and U.S. Mexican families due to limited previous research on this specific type of discourse analysis in ethno-racial minority populations. As a result, this study represents an exploratory effort aimed at expanding the understanding of how frequently guilt and shame inductions are used in real-life interactions within ethno-racial minority populations in the U.S. and investigating the potential consequences associated with these practices.

## Method

### Participants

The study used data from a subsample of the mothers and children who participated in the fifth-grade phase of the Early Head Start Research and Evaluation Project (EHSREP), which was carried out in 17 urban and rural locations across the United States between 2007 and 2009. The final sample included 436 mothers and children (49% girls, 51% boys), representing three distinct ethno-racial groups, including European Americans (*n* = 148), African Americans (*n* = 145) and U.S. Mexicans (*n* = 143). All children were in the fifth grade at the time of data collection (*M*_*age*_ = 10.51, *SD* = 0.52). The selection criteria for our subsample were based on the requirements of the larger project, which specified that participants must have data from three home visits conducted at 24 months, 36 months, and grade 5. Additionally, within the Latine subgroup, we specifically selected U.S. Mexican families due to cultural differences observed among Latine subgroups. Participants who met these requirements from each ethnic group were randomly selected using a random number generator to ensure unbiased representation. Independent samples t-tests were performed on maternal education, family income, children’s externalizing scores, and social adjustment problem scores to assess whether the final sample was representative of the larger dataset. The results indicated no significant differences between the groups.

In accordance with the stipulations of the Early Head Start program, all mothers enrolled in the Early Head Start Research and Evaluation Project (EHSREP) were living at or below the federal poverty line at the time of EHSREP enrollment. During the fifth-grade phase of the EHSREP, the sample’s average household income was $36,264 (*SD* = 30,011) and average income-to-needs ratio was 1.54 (*SD* = 1.31). 27% of mothers did not have high school degrees; 26% had either high school degrees or obtained a GED; 26% had some postsecondary education without a degree; 21% had bachelor’s degrees or higher. 49% of mothers were employed full-time; 20% were employed part-time; and 31% were unemployed. 27% of mothers were living with their husbands; 41% were living with other adults; and 32% were living alone.

## Procedure

During enrollment in the EHSREP and in subsequent data collection waves, demographic information was gathered. In the fifth-grade phase, mothers and children took part in two-hour home visits. Mothers and children were asked to engage in the Parent-Child Discussion Task towards the end of the home visit. They were provided with a set of 15 cards, each featuring a topic that commonly leads to disagreements between parents and children (such as chores, dress choices, school/homework). Children were subsequently instructed to choose three topics from the cards that they wished to discuss with their mother and attempted to find resolutions within an 8-minute timeframe. The discussions were recorded on video. Later on, all the discussions were transcribed from the recorded videotapes by trained research assistants. Transcriptions were meticulously reviewed by a second transcriber for accuracy and quality. To ensure cultural relevance and account for differences in language use across cultures, transcribers were specifically assigned to match the ethnicity of the participating families during the transcription process. Spanish-speaking families’ transcripts were also translated and checked by fluent Spanish speakers. The discussions were then coded by a diverse team, including two European American coders, one Latine coder, and one Asian American coder. In the fifth-grade phase, mothers also completed a measure of children’s internalizing and externalizing behaviors.

### Coding

#### Maternal Use of Guilt and Shame Inductions

Mothers and children’s conflict conversations were coded for maternal use of guilt and shame inductions. The category of guilt induction included maternal statements in which the child was blamed for harming another person, including the mother, through actions such as disrespect, inconvenience, or other means. These statements were designed to make the child feel bad in order to change their behavior. For example, in a discussion about the way the child dresses, one mother stated, “It makes me look bad, like I am not taking care of you. And that is not true.” This statement shifts the focus to the mother’s image, pressuring the child to change their behavior to avoid harming her reputation. This is a clear example of guilt induction, as captured in our coding scheme.

The category of shame inductions included maternal statements in which an aspect of the child’s self is subjected to negative evaluation. This evaluation can pertain to various facets, such as the child’s personality traits, physical appearance, or abilities. For example, one mother criticized her child’s clothing by saying, “You are looking like you just got through mowing grass” and “You do not have to look like hired help.” These statements target the child’s self-image and evoke feelings of shame.

Maternal statements, including both guilt and shame inductions, were systematically coded using a 4-point scale. The coding scheme involved the following interpretations: a code of 0 denoted the absence of any evidence pertaining to guilt/shame references. A code of 1 indicated the presence of a few instances characterized by low intensity guilt/shame references. On the other hand, a code of 2 indicated the occurrence of a few instances of guilt/shame references with moderate intensity, or the frequent emergence of guilt/shame references with low intensity. Finally, a code of 3 reflected either the frequent utilization of guilt/shame references or sporadic instances of guilt/shame references characterized by high intensity. This coding approach allowed for the categorization of the statements according to the varying levels of guilt and shame references present. The coding scheme for maternal use of guilt and shame induction was developed deductively, grounded in a comprehensive and well-established body of literature on psychological control, with particular attention to components related to guilt and shame inductions (e.g., Barber et al., 1994; 2002, 2005, 2012; Fromson, 2006; Fung & Lau, 2012; Rakow et al., 2011; Scarnier et al., 2009; Tangney et al., 1995, 1996, 2014; Vangelisti et al., 1991; Yu et al., 2015). Four trained research assistants were engaged in the systematic coding of maternal guilt and shame inductions. To ensure reliability, a comprehensive coding guideline was developed, and all coders underwent training to align their interpretation and application of the coding criteria. Coders were blinded to participants’ identities and any potentially biasing information to minimize bias. Intraclass correlation coefficients (ICC) were computed to evaluate the reliability of their coding. The results showed a high level of agreement for both maternal guilt inductions (α = 0.96; 1-way ICC, 95% CI [0.93–0.98]) and shame inductions (α = 0.92; 1-way ICC, 95% CI [0.87–0.95]).

### Outcome and Control Measures

#### Externalizing Behavior

Children’s externalizing behavior in the fifth grade was assessed by summing the scores of two subscales of the Child Behavior Checklist for Ages 6–18 (CBCL; Achenbach and Rescorla, 2001): the 18-item Aggressive Behavior Subscale (α = 0.90) and the 17-item Rule-Breaking Behavior Subscale (α = 0.73). The externalizing scale of the Child Behavior Checklist (CBCL) has been extensively employed and has demonstrated strong validation as a reliable measure for assessing behavioral problems (Bub et al., 2007). Mothers rated their children’s aggression levels and rule-breaking behavior on a 3-point Likert scale, ranging from 1 (not true) to 3 (very true). Higher scores indicate a greater presence of externalizing behaviors, such as teasing, screaming, threatening, lying, and truancy.

#### Social Adjustment Problems

To evaluate children’s social adjustment problems in fifth grade, the 11-item Social Problems subscale (α = 0.73) of the Child Behavior Checklist for Ages 6–18 (Achenbach & Rescorla, 2001) was employed. Mothers provided ratings for each item on a 3-point Likert scale, ranging from 1 (not true) to 3 (very true). Higher scores suggest that the child demonstrates a greater frequency of social adjustment problems. This includes difficulty in getting along with peers, experiencing teasing, feeling lonely, displaying behaviors that are not appropriate for their chronological age, and being overly dependent on adults.

#### Maternal Distress

To assess maternal distress, the Parenting Distress subscale of the Parenting Stress Index-Short Form (PSI-SF; Abidin, 1995) was used. This measure evaluates the distress parents experience in their caregiving role, which may stem from factors such as stress related to the perceived demands of parenting, depression, limited social support, and feelings of inadequacy as a parent. The measure includes 5 items, each rated on a 5-point scale. Higher scores indicate higher levels of parental distress (α = 0.73).

### Data Analytic Strategy

The data were analyzed using SPSS Version 29.0 (IBM Corp., 2022) and Mplus Version 8 (Muthén & Muthén, 1998–2017). Missing values in the primary variables were minimal (< 1%), with the highest proportion observed in maternal education (12%), a control variable. Little’s MCAR test was employed to assess the randomness of the missing data. The non-significant result (χ² = 5.66, *p* = .226) supports the assumption that the missing data are completely random. Differences in maternal use of guilt and shame inductions across ethnic/racial groups were analyzed using a one-way analysis of covariance (ANCOVA) in SPSS. Missing data for ANCOVA analyses were addressed using multiple imputation. Additionally, path analysis was performed in Mplus to investigate the associations between maternal use of guilt and shame inductions and children’s externalizing behavior and social adjustment problems. Maternal guilt and shame inductions, as well as children’s externalizing behaviors and social adjustment problems, were allowed to correlate. Additionally, child sex and maternal education were incorporated into the model as statistical controls. Maternal distress was also included as a control variable to account for potential biases in maternal reports of child outcomes. Full information maximum likelihood was applied to handle missing data. Model fit was evaluated using three indices: the comparative fit index (CFI), root-mean-square error of approximation (RMSEA), and standardized root-mean-square residual (SRMR). A model was deemed to have good fit if CFI > 0.90, RMSEA < 0.06, and SRMR < 0.08 (Hu & Bentler, 1999). To examine potential differences in these associations across ethnic/racial groups, a Wald test was conducted.

## Results

### Descriptive Statistics and Correlations

Descriptive statistics, including means and standard deviations for all variables used in this analysis, are displayed in Table 1. Additionally, bivariate correlations were computed to explore the relationships between maternal guilt/shame inductions and children's externalizing behaviors and social adjustment problems. Maternal use of guilt inductions was positively correlated with their use of shame inductions, children’s externalizing behaviors and social adjustment problems. Maternal use of shame inductions was positively correlated with children’s externalizing behaviors and social adjustment problems. Children’s externalizing behavior was positively correlated with their social adjustment problems (Table 1).

**Table 1:** Descriptive statistics and correlations for study variables

Variable	1	2	3	4	5	6
1. Guilt Inductions	1	0.32**	0.19**	0.13**	0.04	− 0.06
2. Shame Inductions		1	0.16**	0.19**	0.07	− 0.06
3. Externalizing Behaviors			1	0.69**	0.04	0.34**
4. Social Adjustment Prob.				1	0.02	0.26**
5. Maternal Education					1	− 0.09
6. Maternal Distress						1
*M*	0.89	0.99	8.48	2.86	11.54	8.34
*SD*	0.80	0.92	8.17	2.87	2.55	3.89

* *p* < .05 * *p* < .01

### Ethnic/Racial Differences in Maternal Use of Guilt and Shame Inductions

**Fig. 1:**
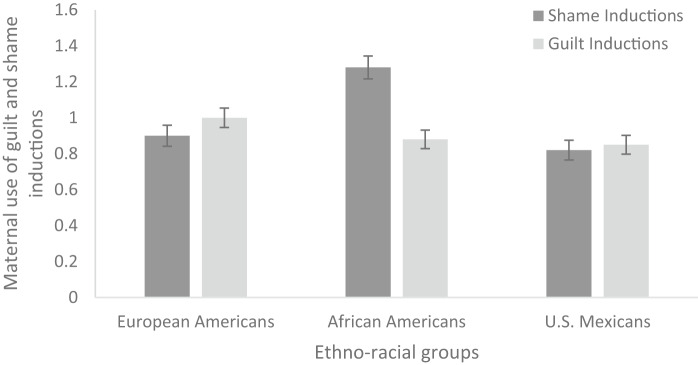
Mean Levels of Maternal Guilt and Shame Inductions by Ethno-Racial Groups

To examine variations among groups regarding maternal utilization of shame inductions, a one-way analysis of covariance (ANCOVA) was conducted. The independent variable was ethnicity/race (three levels: European American, African American, and U.S. Mexican), and the dependent variable was shame induction. Maternal education was used as a covariate. Results revealed significant differences in the amount of shame induction across the three ethno-racial groups after controlling for maternal education, *F* (2, 436) = 7.70, *p* < .001, *η*^*2*^_*p*_ = 0.034. African American mothers (*M* = 1.24, *SD* = 0.94) exhibited a higher use of shame inductions compared to both European American (*M* = 0.91, *SD* = 0.92) and U.S. Mexican mothers (*M* = 0.86, *SD* = 0.87) (see Fig. 1). To examine group differences in maternal use of guilt inductions, another one-way analysis of covariance (ANCOVA) was run with race/ethnicity as the independent variable and guilt induction as the dependent variable. Results showed that there were no differences in the amount of guilt induction across the three ethno-racial groups, *F* (2, 436) = 1.43, *p* = .244, *η*^*2*^_*p*_ = 0.007 (see Fig. 1).

### The Relations between Maternal Guilt and Shame Inductions and Children’s Externalizing Behaviors and Social Adjustment Problems

**Fig. 2:**
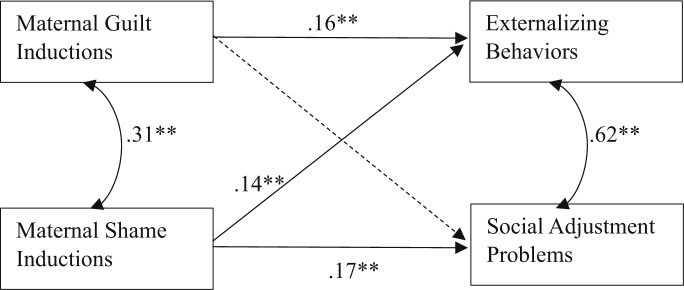
Structural Model Depicting Paths from Maternal Guilt and Shame Inductions to Children’s Externalizing Behaviors and Social Adjustment Problems. Maternal education, child gender, and maternal distress were included as statistical controls. Dashed lines were used to show nonsignificant results. *p < .05 **p < .01

To examine whether maternal use of guilt and shame inductions predicted children’s externalizing behavior and social adjustment problems, path analysis was conducted in Mplus version 8. The hypothesized model demonstrated a good fit to the data, χ^2^ (8) = 5.02, *p* = .755, CFI = 0.99, RMSEA = 0.08, SRMR = 0.02. Maternal guilt inductions were found to have a positive association with children’s externalizing behaviors, *β* = 0.16, *p* = .001, but were not significantly associated with social adjustment problems, *β* = 0.08, *p* = .121 (see Fig. 2). Furthermore, maternal shame inductions showed a positive association both with children’s externalizing behaviors,* β *= 0.14, *p* = .009 and social adjustment problems,* β* = 0.17, *p* = .003.

In order to assess significant differences across racial/ethnic groups, the coefficients for a given path were constrained to be equal across groups. This procedure determined whether the paths were equivalent and identified significant Wald statistics as evidence of significant differences across groups. The model considered externalizing behaviors and social adjustment problems as outcome variables. The results of the Wald test suggested that there were no significant differences in paths across the groups, Wald (4) = 0.396, *p* = .982.

## Discussion

The main aim of this study was to explore how maternal inductions of guilt and shame during conflict conversations relate to children’s externalizing behaviors and social adjustment problems in ethno-racially diverse low-income families. Additionally, the study aimed to examine differences in these patterns among European American, African American, and U.S. Mexican ethno-racial groups. The results revealed that maternal inductions of shame were positively related to both children’s externalizing behaviors and their social adjustment issues. Meanwhile, maternal inductions of guilt were positively related to only externalizing behaviors. Additionally, no significant differences were observed among these ethno-racial groups concerning how guilt and shame inductions relate to externalizing behaviors and social adjustment problems. African American mothers were more likely to use shame inductions compared to European American and U.S. Mexican mothers. However, there were no significant differences between the ethno-racial groups in the use of guilt inductions. These findings underscore the importance of examining the distinct relations of maternal shame and guilt inductions and children’s behavior. Furthermore, the general consistency in how guilt and shame inductions affect children across different ethno-racial groups suggests widespread challenges posed by maternal psychological control for child development in low-income households.

The findings that link maternal inductions of shame with children’s externalizing behaviors and social adjustment problems align well with existing adolescent or parent reports (Ferguson & Stegge, 1998; Tangney et al., 2014; Thomaes et al., 2007). The role of shaming in social adjustment issues could be multifaceted, potentially arising from increased sensitivity towards others’ criticism or from diminished self-esteem resulting from the experience of being shamed. This can cause children to become overly cautious or less confident in social interactions, which may lead to poor interpersonal relations (Eisenberg, 2000; Leith & Baumeister, 1998). Moreover, shaming can prompt externalizing behaviors to protect self-esteem and regain autonomy (Tangney et al., 2014). Thus, externalizing behaviors may be considered attempts to externally redirect the internal discomfort caused by shame. This behavioral response not only underscores the role of shaming as a significant stressor but also highlights its counterproductive outcome in social and behavioral development. Additionally, these findings could be the result of a cyclical effect where shaming exacerbates the very behaviors it often aims to curb, thus reinforcing negative patterns within parent-child interactions.

The finding that maternal inductions of guilt are associated with children’s externalizing behaviors adds depth to existing research, which has already established the link between general psychological control and such behaviors (Bai et al., 2020; Gugliandolo et al., 2015; Symeou & Georgiou, 2017). This finding reinforces the idea that guilt induction, akin to general psychological control, can increase anger in children, which may then show up as externalizing behaviors. The study calls for detailed research into the underlying mechanisms by which induced feelings of guilt may interfere with a child’s ability to regulate emotions and potentially lead to externalizing behaviors.

However, contrary to what we anticipated, guilt inductions were not found to be associated with social adjustment problems. Although guilt inductions are associated with externalizing behaviors, they may not directly impair a child’s social functioning or their ability to interact with peers. This may be partially attributed to the nature of guilt inductions themselves, which typically focus on specific actions rather than criticizing the child’s overall character (Tangney et al., 1996). This specificity may prevent guilt from severely undermining a child’s self-image or social competence. Also, social adjustment encompasses a range of complex skills such as adaptability, problem-solving, and negotiation, which may develop through a variety of interactions beyond the family (Caldarella & Merrell, 1997) and may not be directly influenced by feelings of guilt. The specific focus on maternal guilt induction in the present study underscores its complex role within the spectrum of psychological control tactics used by mothers. It highlights how guilt induction is related to child behavior in nuanced ways, affecting externalizing behaviors while leaving broader social skills and adjustment relatively intact. This distinction invites further investigation into the differential roles of various forms of psychological control in child development.

Contrary to our initial hypotheses, our study found no significant differences among ethno-racial groups regarding the association between guilt and shame inductions and children’s social adjustment and externalizing problems. This result aligns with previous research indicating that the links between these parental practices and child behavioral problems are consistent across diverse ethno-racial backgrounds (Barber et al., 2005; Kincaid et al., 2011; Plunket et al., 2016; Soenens et al., 2012), but it extends to low-income households. These findings might reflect a strong need for autonomy across ethno-racial groups and suggest that guilt and shame inductions can threaten this autonomy (Soenens & Vansteenkiste, 2010). Mixed findings observed in previous studies among ethnic-racial groups may be linked to the use of culturally non-sensitive, self-report measures designed to assess psychological control. The use of an observational design in the present study allowed for the direct observation of real-life interactions between mothers and children and offered a valuable perspective for examining the association between maternal guilt and shame inductions and child behavioral outcomes across diverse ethno-racial families. Alternatively, the findings could also result from the conflict focus of the mother-child conversations, which could skew discourse towards stricter psychological control techniques. This latter possibility raises questions about how the content or context of psychological control may amplify or mitigate its negative effects. 

Lastly, the higher frequency of shame inductions observed among African American mothers compared to both European American and U.S. Mexican mothers partly aligns with prior research indicating that African American parents are more likely to use authoritarian parenting strategies in contrast to European American parents (LeCuyer & Swanson, 2017; Ng et al., 2014). It is crucial to contextualize African American mothers’ use of shame inductions. By employing psychological control practices, African American mothers may seek to guide their children’s behavior in ways that help mitigate the detrimental effects of systemic inequalities and racism. Moreover, African American women are often exposed to chronic stressors linked to racial, social, and economic disadvantage (APA, 2017). As suggested by the Family Stress Model, such exposure can lead to maternal distress, which negatively influences parental functioning and the quality of parent-child interactions (Conger et al., 2010). Consistent with this model, prior work has shown that higher parental stress is associated with greater use of intrusive or harsh parenting, increased conflict, and reduced closeness and enjoyment in parent-child interactions (Chung et al., 2022; Crnic et al., 2005; Ispa et al., 2004). Thus, the higher frequency of shame inductions employed by African American mothers should be interpreted within the distinctive sociopolitical context characterized by unique stressors that shape parenting in African American communities. These practices may reflect broader adaptive strategies aimed at fostering resilience and preparing children for the societal challenges they are likely to face. 

### Strengths, Limitations, and Implications for Future Studies

Our study holds several strengths that contribute to its novel insights. First, this study utilized observational coding of psychological controlling discourse, which is a more objective method compared to relying solely on child or parent self-reports of guilt and shame inductions, as seen in previous studies. This approach enabled us to directly observe and analyze the actual messages sent by mothers, thus providing a clearer picture of mother-child interactions. This methodological choice strengthens the validity of our results and supports a more nuanced understanding of the associations between maternal guilt and shame inductions and children's behavioral outcomes across diverse ethno-racial groups. To our knowledge, this study stands as the first cross-cultural examination of maternal guilt and shame inductions among European American, African American, and U.S. Mexican families, which allows direct comparisons across these groups. An additional strength is the exclusive focus on a low-income sample. This choice enables a comprehensive exploration of the associations between maternal psychological control and child outcomes in a diverse sample, while effectively controlling for the influence of socioeconomic status.

The limitations of the current study highlight the need for future research. Due to the cross-sectional design, causal inferences and the direction of relationships cannot be drawn from this study. Conducting future longitudinal studies may offer stronger evidence regarding the direction of the relationships between maternal guilt and shame inductions and children’s problem behaviors. Furthermore, the sample may reflect a limited representation of families within the included ethno-racial groups. Future studies may expand the representation of ethno-racial groups in their samples to better capture the cultural diversity related to maternal psychological control and children’s behavioral issues. Moreover, although externalizing behaviors were relatively low in the sample, potentially constraining variability in the outcome measure, significant associations with guilt and shame inductions were still observed. However, the limited range of externalizing behaviors may restrict generalizability, particularly to clinical or high-risk populations with more prevalent behavior problems. Future research with more diverse samples and a wider distribution of behavioral ratings is needed to validate these results. Another limitation of this study is the reliance on archival data, which may not fully capture recent shifts in the topics of conflict or parenting practices. However, the core dynamics of parent-child communication and psychological control remain relevant, as they continue to influence relationships and outcomes across diverse cultural contexts.

Despite the inherent limitations, the present findings contribute to understanding the diverse associations between maternal shame and guilt inductions and children’s problem behaviors. These patterns may indicate that separate strategies might be needed to address these problem behaviors. Interventions targeting shame inductions could be particularly useful in improving children’s social adjustment and reducing externalizing behaviors. Similarly, efforts to mitigate maternal guilt inductions may specifically help in decreasing externalizing behaviors in children. The uniformity in the relations between guilt and shame inductions and children’s behavior across various ethno-racial groups could highlight the common difficulties that maternal psychological control presents in child development. Future studies could expand to include additional ethno-racial groups to determine if these patterns hold true universally or if unique cultural factors might modify these associations. Additionally, future research efforts could focus on identifying the factors that explain the underlying mechanisms of the associations between maternal guilt and shame inductions and child outcomes. By elucidating the mechanisms through which maternal discourse styles influence child behavior, we can refine and enhance our parenting interventions and ultimately foster positive developmental outcomes for children.
